# Longitudinal lung function trajectories in response to azithromycin therapy for chronic lung disease in children with HIV infection: a secondary analysis of the BREATHE trial

**DOI:** 10.1186/s12890-024-03155-x

**Published:** 2024-07-12

**Authors:** Tafadzwa Madanhire, Grace McHugh, Victoria Simms, Lucky Ngwira, Carmen Gonzalez-Martinez, Robina Semphere, Brewster Moyo, Claire Calderwood, Mark Nicol, Tsitsi Bandason, Jon O. Odland, Andrea M. Rehman, Rashida A. Ferrand

**Affiliations:** 1 The Health Research Unit Zimbabwe, Biomedical Research & Training Institute, 10 Seagrave Road, Harare, Zimbabwe; 2 MRC International Statistics and Epidemiology Group, London School of Hygiene & Tropical Medicine, London, UK; 3 Malawi-Liverpool-Wellcome Trust Clinical Research Programme, Blantyre, Malawi; 4 Department of Paediatrics and Child Health, University of Malawi College of Medicine, Blantyre, Malawi; 5 Division of Medical Microbiology, Department of Pathology, Faculty of Health Sciences, University of Cape Town, Cape Town, South Africa; 6 Division of Infection and Immunity, School of Biomedical Sciences, University of Western Australia, Perth, Australia; 7 Faculty of Health Sciences, UiT, The Arctic University of Norway, Tromsø, Norway; 8 School of Health Systems and Public Health, Faculty of Health Sciences, University of Pretoria, Pretoria, South Africa; 9 Clinical Research Department, London School of Hygiene and Tropical Medicine, London, UK

**Keywords:** Chronic lung disease, HIV, FEV_1_, Africa, Azithromycin, Children, Adolescents

## Abstract

**Background:**

Chronic lung disease (CLD) is common among children with HIV (CWH) including in those taking antiretroviral therapy (ART). Azithromycin has both antimicrobial and anti-inflammatory effects and has been effective in improving lung function in a variety of lung diseases. We investigated lung function trajectories among CWH with CLD on ART enrolled in a randomized controlled trial of adjuvant azithromycin. We also investigated factors that modified the effect of azithromycin on lung function.

**Methods:**

The study used data from a double-blinded placebo-controlled trial conducted in Malawi and Zimbabwe of 48 weeks on azithromycin (BREATHE: ClinicalTrials.gov NCT02426112) among CWH aged 6 to 19 years taking ART for at least six months who had a forced expiratory volume in one second (FEV_1_) z-score <-1.0. Participants had a further follow-up period of 24 weeks after intervention cessation. FEV_1_, forced vital capacity (FVC) and FEV_1_/FVC were measured at baseline, 24, 48 and 72-weeks and z-scores values calculated. Generalized estimating equations (GEE) models were used to determine the mean effect of azithromycin on lung-function z-scores at each follow-up time point.

**Results:**

Overall, 347 adolescents (51% male, median age 15 years) were randomized to azithromycin or placebo. The median duration on ART was 6.2 (interquartile range: 3.8–8.6) years and 56.2% had an HIV viral load < 1000copies/ml at baseline. At baseline, the mean FEV_1_ z-score was − 2.0 (0.7) with 44.7% (*n* = 155) having an FEV_1_ z-score <-2, and 10.1% had microbiological evidence of azithromycin resistance. In both trial arms, FEV_1_ and FVC z-scores improved by 24 weeks but appeared to decline thereafter. The adjusted overall mean difference in FEV_1_ z-score between the azithromycin and placebo arms was 0.004 [-0.08, 0.09] suggesting no azithromycin effect and this was similar for other lung function parameters. There was no evidence of interaction between azithromycin effect and baseline age, lung function, azithromycin resistance or HIV viral load.

**Conclusion:**

There was no observed azithromycin effect on lung function z-scores at any time point suggesting no therapeutic effect on lung function.

**Trial registration:**

ClinicalTrials.gov NCT02426112. First registered on 24/04/2015.

**Supplementary Information:**

The online version contains supplementary material available at 10.1186/s12890-024-03155-x.

## Background

Worldwide, an estimated 2.8 million children and adolescents (0–19 years) live with HIV, with 1.8 million in eastern and southern Africa [1]. The roll-out of antiretroviral therapy (ART) has improved survival of children with HIV (CWH), so that growing numbers are reaching adolescence and adulthood. There is a growing evidence however, that despite ART, CWH experience a range of multisystem chronic comorbidities [2].

Studies in recent years have shown that up to a third of African CWH have chronic lung disease (CLD) typically with cough, hypoxia and significantly reduced exercise tolerance and impaired lung function. Radiological studies are consistent with the aetiology being constrictive obliterative bronchiolitis (COB) [3]. The lack of association between abnormal lung function and ART use and duration or CD4 count, suggests that this form of HIV-related CLD may not be responsive to ART once established [4, 5]. While the underlying drivers of COB in the context of HIV infection are not well understood, it is thought to be a consequence of chronic inflammation either due to HIV-mediated aberrant systemic immune activation or recurrent lung co-infections (which children with HIV are at elevated risk of), with tissue injury followed by aberrant fibro-proliferative remodelling in the small airways [6–12]

Azithromycin is an oral macrolide that has broad-spectrum antibiotic properties and is used to treat a wide range of infections including respiratory tract infections [13]. Azithromycin is also recognised to have immunomodulatory properties [14]. It likely reduces inflammation and has been shown to have therapeutic effects in several chronic lung diseases including improvement in lung function and reduction in infective exacerbations in patients with cystic fibrosis, [15] and improvement in forced expiratory volume in one second (FEV1) in obliterative bronchiolitis syndrome in the context of lung transplantation [16–18].

We hypothesized that azithromycin may therefore also improve lung function and reduce risk of infective exacerbations among children with HIV-associated CLD, through its immunomodulatory and antibiotic properties respectively [19]. We conducted a double-blind, placebo-controlled individually randomised trial (BREATHE) to investigate the impact of 48 weeks of weekly azithromycin on lung function and risk of acute respiratory exacerbations (ARE) in older children with HIV. The main trial results including the safety profile of azithromycin have been published previously [20]. In this secondary analysis, we investigated the effect of adjuvant azithromycin therapy on lung function trajectories (FEV1, forced vital capacity [FVC] and FEV1/FVC z-score) among CWH on ART with CLD and whether any treatment effects persist after cessation of treatment. We also investigated factors that could modify potential therapeutic effects of azithromycin on lung function.

## Methods

### Study design and setting

Participants were enrolled between June 2016 and September 2018 into the BREATHE trial (ClinicalTrials.gov NCT02426112) from public sector HIV clinics in Blantyre, Malawi and Harare, Zimbabwe, with a follow-up period of up to 72 weeks [20, 21].

### Trial procedures

The trial protocol has been published [20, 21]. Briefly, eligibility criteria were age 6–19 years, having perinatally acquired HIV, being on ART for at least six months and an FEV_1_ z-score < -1.0. Other eligibility criteria were having been disclosed their HIV status (for those aged ≥ 12 years) and having a guardian able to provide consent (for those aged < 18 years).

Exclusion criteria were having a condition likely to be fatal during the study period (e.g. malignancy), acute respiratory tract infection or tuberculosis (screened for using the Xpert™ MTB/RIF (Cepheid, Sunnyvale, CA, USA) on one sputum sample), pregnancy or breastfeeding, history of cardiac arrhythmia, a prolonged QTc interval (> 440 and > 460 milliseconds in males and females respectively), creatinine clearance < 30mls/minute, alanine aminotransferase > 2 times the upper limit of normal, known macrolide hypersensitivity, or use of drugs known to prolong the QTc interval.

Participants were randomised to either receive an oral weekly weight-based dose of azithromycin or placebo for 48 weeks. Following enrolment, participants were followed up at 2, 12, 24 and 36 weeks for ascertainment of incident symptoms, side-effects and adherence and for drug re-supply.

Lung function parameters (FEV_1_, FVC and FEV_1_/FVC) were measured by spirometry (with American Thoracic Society quality assessment criteria met pre bronchodilator) at enrolment, 24, 48 and at 72 weeks. HIV viral load was also assessed at enrolment and 48 weeks. All participants were tested for tuberculosis with a sputum sample for Xpert™ MTB/RIF (Cepheid, Sunnyvale, CA, USA) at baseline. Azithromycin sensitivity profiles were assessed using the Kirby–Bauer disk diffusion method.

The trial had 80% statistical power to detect a standardized mean difference in FEV_1_ z-score of 0.32 SD between the two trial arms at a 95% confidence level at 48 weeks.

### Statistical analysis

Data analysis was performed using STATA 16 (StataCorp College Station, TX). Measurements were included in analysis if obtained within 4 weeks either side of the scheduled appointment. The corresponding z-scores of the lung function parameters were calculated using GLI African American spirometric reference equations adjusting for age, sex, height and ethnicity [22, 23].

Participant demographics and clinical assessments were summarized at all time points, reporting means (with standard deviation (SD)) and proportions (with % values) for continuous and categorical variables respectively. Mean and 95% confidence interval trajectories for FEV_1_, FVC and FEV_1_/FVC z-scores were visualized to show crude longitudinal trends over time.

A marginal mean regression model of the form Y_t_ = β_0_ + β_1_X + β_2_Y_t0_ + β_3_time + β_4_X* time (where Y_t_ is the lung function z-score outcome measured at three follow-up time points and X is the treatment variable) that considered the time of follow-up and trial arm was developed using the Generalized Estimating Equation (GEE) model, with robust-standard errors and an unstructured covariance structure [24]. The treatment effects at 24, 48 and 72 weeks were considered as β_1_ and β_1_ + β_4_ respectively. Further model adjustments to include factors that were imbalanced by trial arm at baseline were made. An interaction term between treatment and time variables was introduced in the model to constrain no effect for trial arm at baseline [25].

Subgroup analysis for the relationship between the intervention and lung function z-scores were performed in pre-specified sub-groups including (i) baseline severity of lung disease (<-2 vs. ≥-2 z-score) and (ii) baseline viral load (< 1000 copies/ml vs. > = 1000 copies/ml) (iii) age group (< 11 years vs. 11–16 years vs. ≥ 16 years) and (iv) carriage of azithromycin-resistant bacteria. Effect modification was examined by incorporating an interaction term between subgroups, trial arm and time of follow-up.

As a sensitivity analysis, we compared participant characteristics at baseline for those who completed follow up vs. those were lost to follow-up. In addition, multiple imputation with 10 iterations was used to impute viral load (natural log time non-varying variable) at 48-week timepoint where it was missing (5%). The registered variables associated with viral load missingness were baseline viral load, sex, age, weight and height.

## Results

**Fig. 1:**
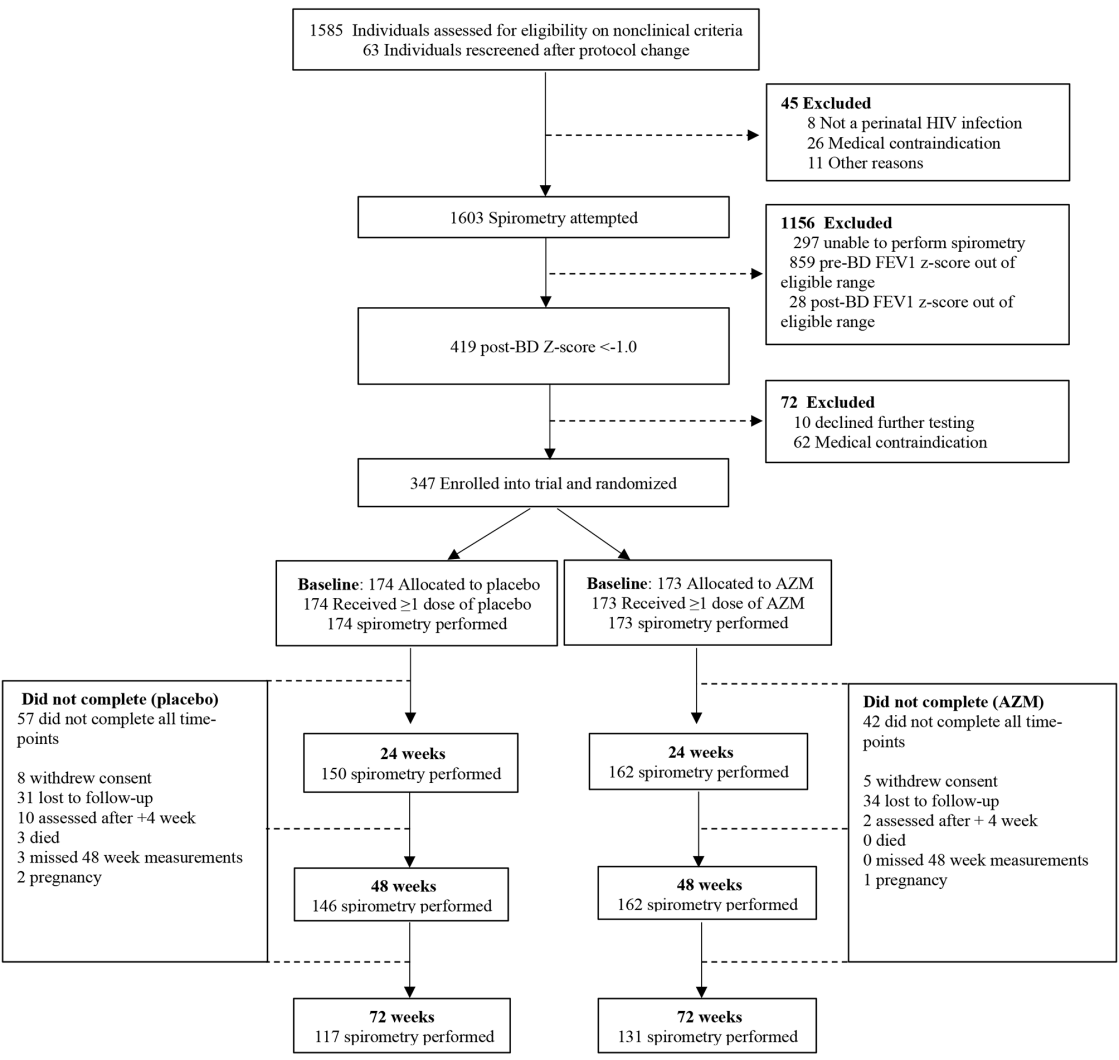
Participant enrolment flow diagram

In total, 347 (51% (*n* = 177) male) participants were recruited of whom 308 (88.8%) were retained in follow-up by 48 weeks and 248 (71.5%) by 72 weeks. More participants in the placebo arm (*n* = 54/174) were lost to follow-up as compared to intervention arm (*n* = 39/173) during the study period (Fig. 1).

### Participant characteristics at baseline

Median age was 16.7 (IQR:14.2–19.2] years and participants had been taking ART for a median 6.2 (IQR: 3.8–8.6) years, with 55.9% of participants having a VL < 1000copies/ml at enrolment. Almost half (175/347) of participants had stunting (defined as height for age z-score <-2) and underweight (181/347: defined as weight for age z-score <-2). Furthermore, 44.7% of participants had an FEV_1_ z-score <-2. The distribution of participants with an FEV_1_ z-score <-2 were balanced between the two arms although mean absolute FEV_1_ values were lower in the azithromycin group [mean: 1.59 (SD: 0.50)] than placebo [mean: 1.71 (SD: 0.53). Overall, no participant had a sputum positive TB culture and one in 10 (35/347) participants were azithromycin resistant at baseline (Table 1). In addition, baseline characteristics were similar between participants who completed follow up (*n* = 248) vs. those who were lost to follow up (*n* = 99) (Supplementary Table 1).

**Table 1:** Characteristics of participants at baseline by trial arm

Variables	AZM (*N* = 173)	Placebo (*N* = 174)
**Demographic characteristics, n (%)**		
Age categories at baseline		
<11 years 11–15 years ≥16 years	25 (14.5) 87 (50.3) 61 (35.3)	22 (12.6) 73 (42.0) 79 (45.4)
Sex		
Male Female	93 (53.8) 80 (46.2)	84 (48.3) 90 (51.7)
Country		
Malawi Zimbabwe	53 (30.6) 120 (69.4)	53 (30.5) 121 (69.5)
**Clinical history**		
Viral load (< 1000 copies/ml), n (%)	100 (58.5)	94 (54.0)
Duration taking ART, median (IQR)	5.9 (3.8-9.0)	6.4 (3.9–8.2)
**Anthropometry**,** n (%)**		
Height for age		
<-2 ≥-2	95 (54.9) 78 (45.1)	80 (46.0) 94 (54.0)
Weight for age		
<-2 ≥-2	98 (56.7) 75 (43.4)	83 (47.7) 91 (52.3)
**Spirometry**,** mean (SD)**		
FEV_1_	1.59 (0.50)	1.71 (0.53)
FEV_1_ z-score	-2.01 (0.76)	-2.0 (0.74)
FVC,	1.89 (0.59)	2.04 (0.63)
FVC z-score	-1.77 (0.97)	-1.71 (0.89)
FEV_1_/FVC	0.85 (0.08)	0.84 (0.08)
FEV_1_/FVC z-score	-0.66 (1.14)	-0.74 (1.13)
**Severity of lung disease**,** n (%)**		
FEV_1_ z-score		
<-2 ≥-2	78 (45.1) 95(54.9)	77 (44.3) 97 (55.7)
FVC z-score		
<-2 ≥-2	61 (35.9) 109 (64.1)	54 (32.0) 115 (68.1)
FEV_1_/FVC z-score <-2 ≥-2	18 (10.6) 152 (89.4)	22 (13.0) 147 (87.0)
Resistance to AZM	15 (8.7)	20 (11.5)

AZM: Azithromycin; IQR: Interquartile range; 4 and 2 missing values for FVC and viral load respectively

### Crude spirometry measurements at follow-up (24, 48 and 72 weeks)

**Fig. 2:**
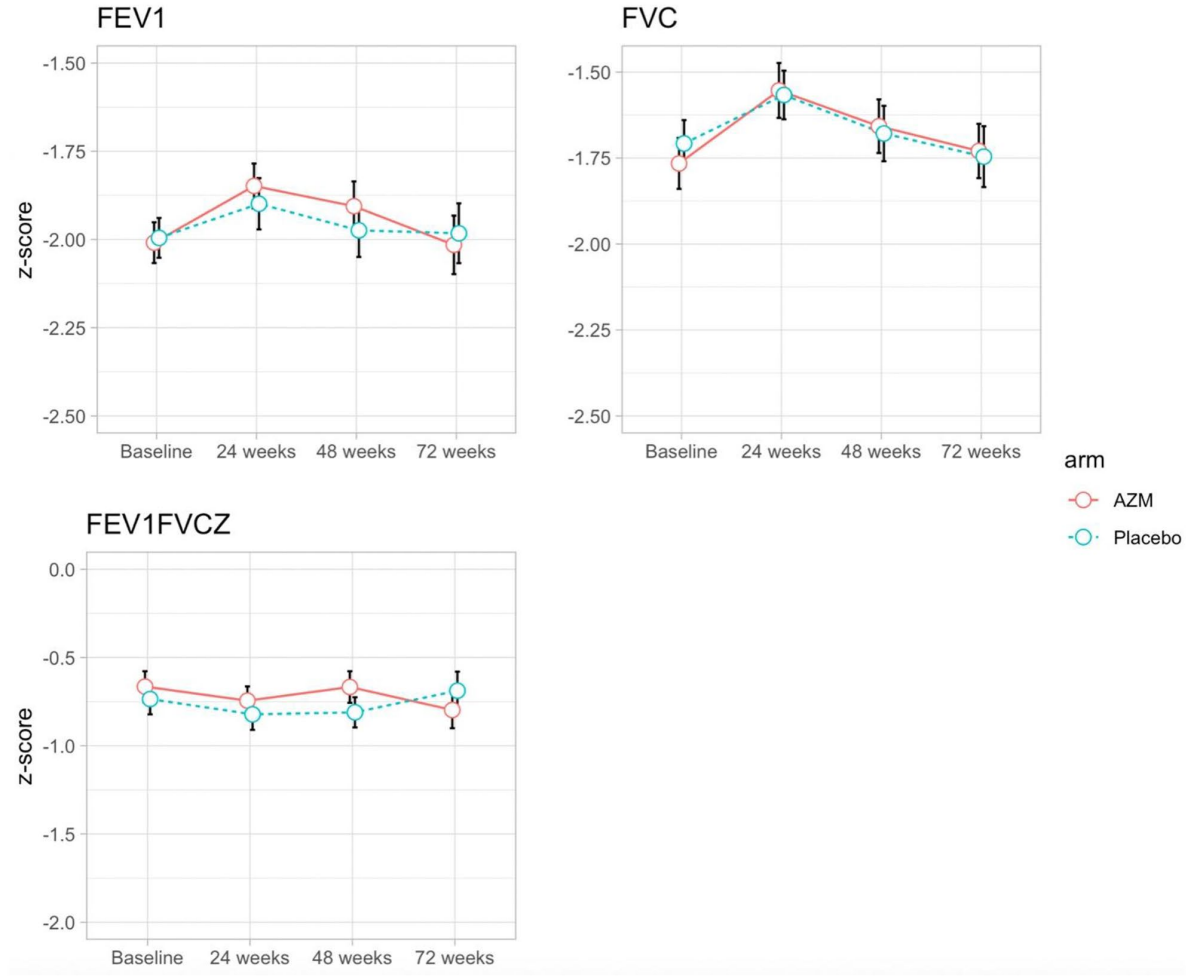
Unadjusted mean scores and 95% confidence intervals for spirometry measurements at 24, 48 and 72 weeks by treatment arm

In both arms, absolute FVC and FEV_1_ measures showed improvements over the follow-up period (Supplementary Fig. 1). In both arms, the corresponding z-score values showed an increase from baseline to 24 weeks (FVC; p value: 0.021, FEV_1_; p value: 0.037 [Fig. 2]). There was a 5% and 3% reduction in the proportion of children with a z-score < -2 between baseline and 24 weeks for FEV_1_ and FVC respectively regardless of arm. However, FEV_1_ and FVC z-scores in both arms at 48 and 72 weeks were comparable to baseline measurements (Fig. 2). There was no change over time in FEV_1_/FVC ratio in either arm.

### Adjusted lung function trajectories by trial arm

In z-score models adjusted for sex, age, site and natural log HIV viral load, there was no statistical evidence for a treatment effect at 24 weeks, with a mean difference between the intervention and control arm in FEV_1_ of -0.02 (95% CI: -0.05, 0.02), in FVC of -0.03 [95% CI: -0.09, 0.03] and in FEV_1_/FVC ratio of 0.02 (95% CI: -0.04, 0.08; Table 2). At 48 and 72 weeks, both adjusted FEV_1_ (48 weeks: 0.02 [95% CI: -0.04, 0.09]; 72 weeks: 0.03 [95% CI: -0.05, 0.10]) and FVC (48 weeks: 0.04 [95% CI: -0.05, 0.13]; 72 weeks: 0.04; [-0.06, 0.13]) z-scores showed a weak positive azithromycin treatment effect, however there was no statistical evidence for a treatment effect. There was no evidence of azithromycin treatment effect on FEV_1_/FVC z-score at either 48 weeks [-0.01; 95% CI: -0.11, 0.09] or 72 weeks [-0.03; 95% CI: -0.07, 0.14]. Overall, there was no treatment effect on lung function z-scores after factoring the changes across the individual time points; FEV_1_: 0.004 [95% CI: -0.08, 0.09], FVC: 0.02 [95% CI: -0.07, 0.12] and FEV_1_/FVC: -0.01 [95% CI: -0.12, 0.09]. Similarly, all adjusted models of absolute lung function measurements showed no evidence of azithromycin treatment effect in crude lung measurements at all time points (Table 2). In the sensitivity analyses, results were similar to unimputed data.

**Table 2:** Treatment effect (adjusted mean difference) for spirometry variables at 24, 48 and 72 weeks

Outcome	Adjusted mean difference (95% CI)			
	24 weeks (*n* = 312)	48 weeks (*n* = 308)	72 weeks (*n* = 248)	Overall effect
FEV_1_	-0.12 [-0.31, 0.07]	0.04 [-0.01, 0.09]	0.05 [-0.07, 0.16]	0.004 [-0.08, 0.09]
FEV_1_ z-score	-0.02 [-0.05, 0.02]	0.02 [-0.04, 0.09]	0.03 [-0.05, 0.10]	0.004 [-0.08, 0.09]
FVC	-0.19 [-0.51, 0.12]	0.04 [-0.02, 0.09]	0.06 [-0.07, 0.19]	0.03 [-0.02, 0.09]
FVC z-score	-0.03 [-0.09, 0.03]	0.04 [-0.05, 0.13]	0.04 [-0.06, 0.13]	0.02 [-0.07, 0.12]
FEV_1_/FVC	0.02 [-0.03, 0.08]	0.01 [-0.01, 0.02]	-0.01 [-0.03, 0.02]	0.005 [0.01, 0.02]
FEV_1_/FVC z-score	0.02 [-0.04, 0.08]	-0.01 [-0.11, 0.09]	0.03 [-0.07, 0.14]	-0.01 [-0.12, 0.09]

The models shows adjusted mean differences between the intervention and placebo arms with negative values indicating lower values in the intervention arm as compared to the placebo. The xtgee models were adjusted for sex, age, site and natural log HIV viral load

### Stratification of models by lung function severity, age, drug resistance and viral load

**Fig. 3:**
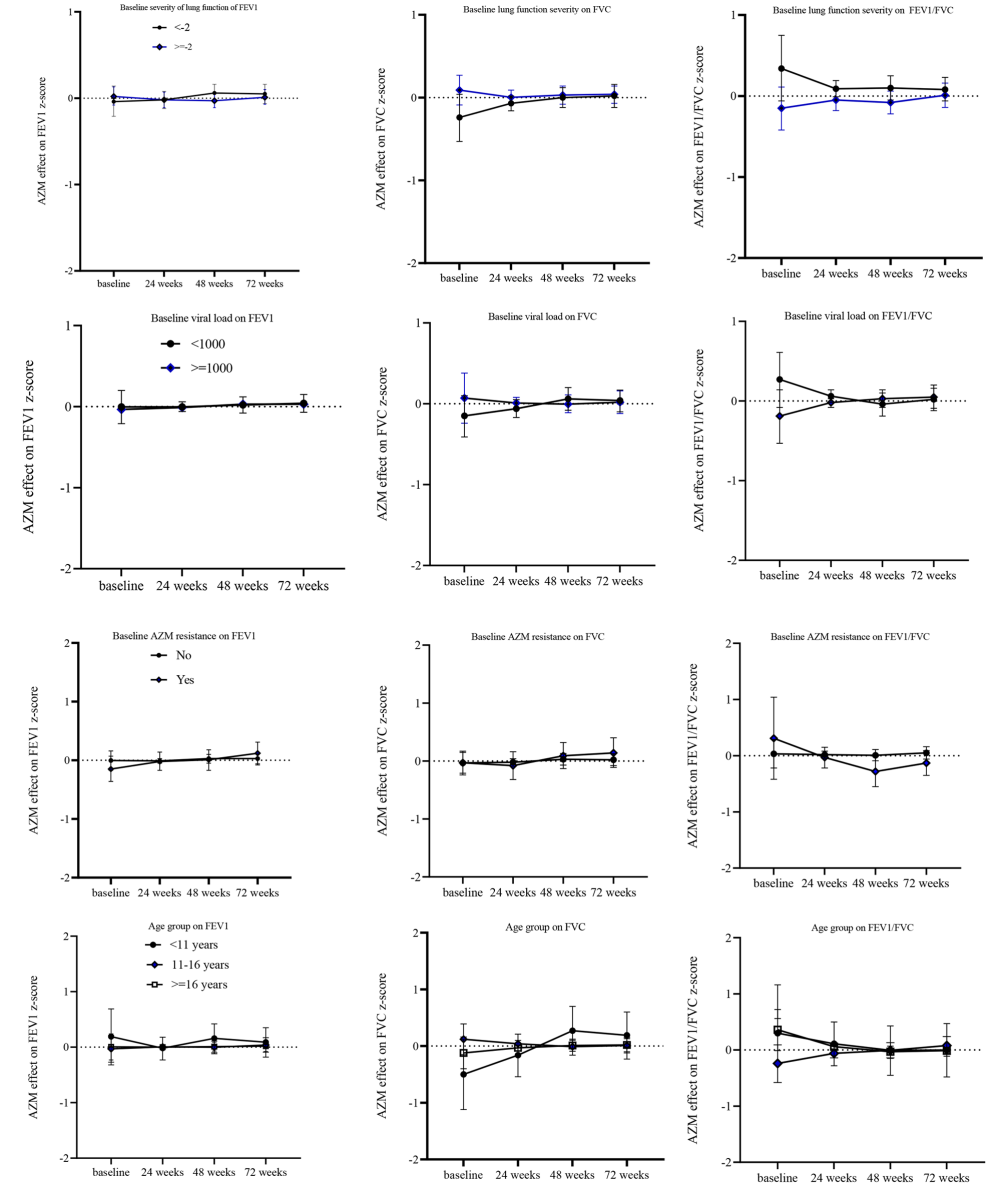
Interaction between AZM with baseline lung function severity, viral load, AZM resistance and age group on lung function outcomes

Further adjustments on fully adjusted FEV_1_, FVC and FEV_1_/FVC z-score models showed no interaction of azithromycin treatment by baseline lung function severity, baseline viral load, age and azithromycin drug resistance on lung function (Fig. 3).

### Safety

There were no severe adverse events related to the study medication [20]. (Supplementary Table 2).

## Discussion

This study investigated the trajectories of lung function in CWH. Overall, the study showed no improvements in any lung function parameter (FEV_1_, FVC and FEV_1_/FVC z-scores) after 48 weeks of azithromycin.

Studies have investigated azithromycin therapy in a number of chronic lung diseases with discrepant findings. In a study of patients with non-cystic fibrosis bronchiectasis, azithromycin did not result in significant improvements in FEV_1_ or FVC compared to placebo [26]. A randomised controlled trial in individuals with chronic obstructive pulmonary disease showed no differences in lung function parameters between azithromycin-treated and placebo groups [27]. Other studies have reported improvements in lung function parameters [28], reduced respiratory symptoms, [29] and decreased rate of exacerbations in various chronic lung diseases conditions with azithromycin. In a meta-analysis of ten studies of patients with bronchiolitis obliterans syndrome (BOS), azithromycin was shown to improve FEV_1_ [30]. Also, longitudinal meta-analysis of studies among cystic fibrosis patients reported gains in percentage predicted FEV_1_ and FVC values with azithromycin [31, 32]. These studies suggest that azithromycin’s anti-inflammatory and immunomodulatory properties may contribute to its beneficial effects on lung function. It should be noted that none of these studies were conducted among individuals with HIV infection.

Our study found azithromycin supplementation had no effect on FEV_1_/FVC z-scores for participants with either severe lung or mild lung function impairment. One study in the United States identified patients with moderately impaired lung function to benefit more than the severely ill ones though airway microbiology was not systematically evaluated in the study [27, 33]. Similarly, in a study to understand the effect of low-dose azithromycin therapy on acute exacerbations of chronic obstructive pulmonary disease (COPD), the study identified improvement in lung function among those with severe exacerbations in the previous year [34].

The lack of effect may be due to the fact that the lung injury in HIV-associated chronic lung disease, once established is not reversible. In constrictive obliterative bronchiolitis, inflammation is followed by fibrosis occurring predominantly in the walls and contiguous tissues of membranous and respiratory bronchioles, with resultant narrowing of their lumens [35]. It may be that at the stage that treatment was started, most children have already developed fibrosis which may not be amenable to immunomodulatory therapy.

Another possible reason for the lack of effect on lung function is insufficient levels of adherence. We have previously reported that adherence was higher for those randomised to AZM (73.4%) than placebo (68.4%) and declined over the 48 weeks of the study (Score test for trend < 0.02). Those with unsuppressed HIV viral load at baseline had 2.08 (95% CI: 1.19, 3.63) times the odds of non-adherence than those with viral suppression [36]. A statistically significant effect of azithromycin on risk of acute respiratory exacerbations was however, observed in the BREATHE trial, with a 50% reduction in the risk of ARE. The impact of azithromycin on reducing AREs was greater in participants with chronic respiratory symptoms at baseline, those on 1st line ART, with a FEV_1_ score >-2 and participants without baseline resistance to azithromycin [29].

The study used data from a well-powered clinical trial and spirometry was performed to ATS standards with inbuilt quality control, and microbiological azithromycin resistance data were available. We had long-term follow-up data enabling tracking of longitudinal trajectories of lung function. Note that by 72 weeks, 28.5% were lost to follow-up, reducing statistical power.

In summary, our study showed no beneficial effect of azithromycin supplementation on lung function in CHW who had CLD, overall or in any subgroup.

### Electronic supplementary material

Below is the link to the electronic supplementary material.

Supplementary Material 1

Supplementary Material 2

## Data Availability

The datasets used and/or analysed during the current study are available at the London School of Hygiene and Tropical Medicine repository (Data Compass) on reasonable request to the corresponding author.

## Abbreviations

HIV: Human Immunodeficiency Virus
ART: Antiretroviral Therapy
CWH: Children living with HIV
CLD: Chronic Lung Disease
OB: Obliterative Bronchiolitis
FVC: Forced Vital Capacity
FEV_1_: Forced Expiratory Volume in 1 s
COPD: Chronic Obstructive Pulmonary Disease
GLI: Global Lung Function Initiative
GEE: Generalized Estimating Equation
