# Supplementary material for: Longitudinal lung function trajectories in response to azithromycin therapy for chronic lung disease in children with HIV infection: a secondary analysis of the BREATHE trial
Source: BMC Pulm Med. 2024 Jul 12;24:339. doi: 10.1186/s12890-024-03155-x (PMC11245797; doi:10.1186/s12890-024-03155-x)

**Supplementary Figure 1: The distribution of crude lung function measurements and their respective z-scores over the follow up time.**


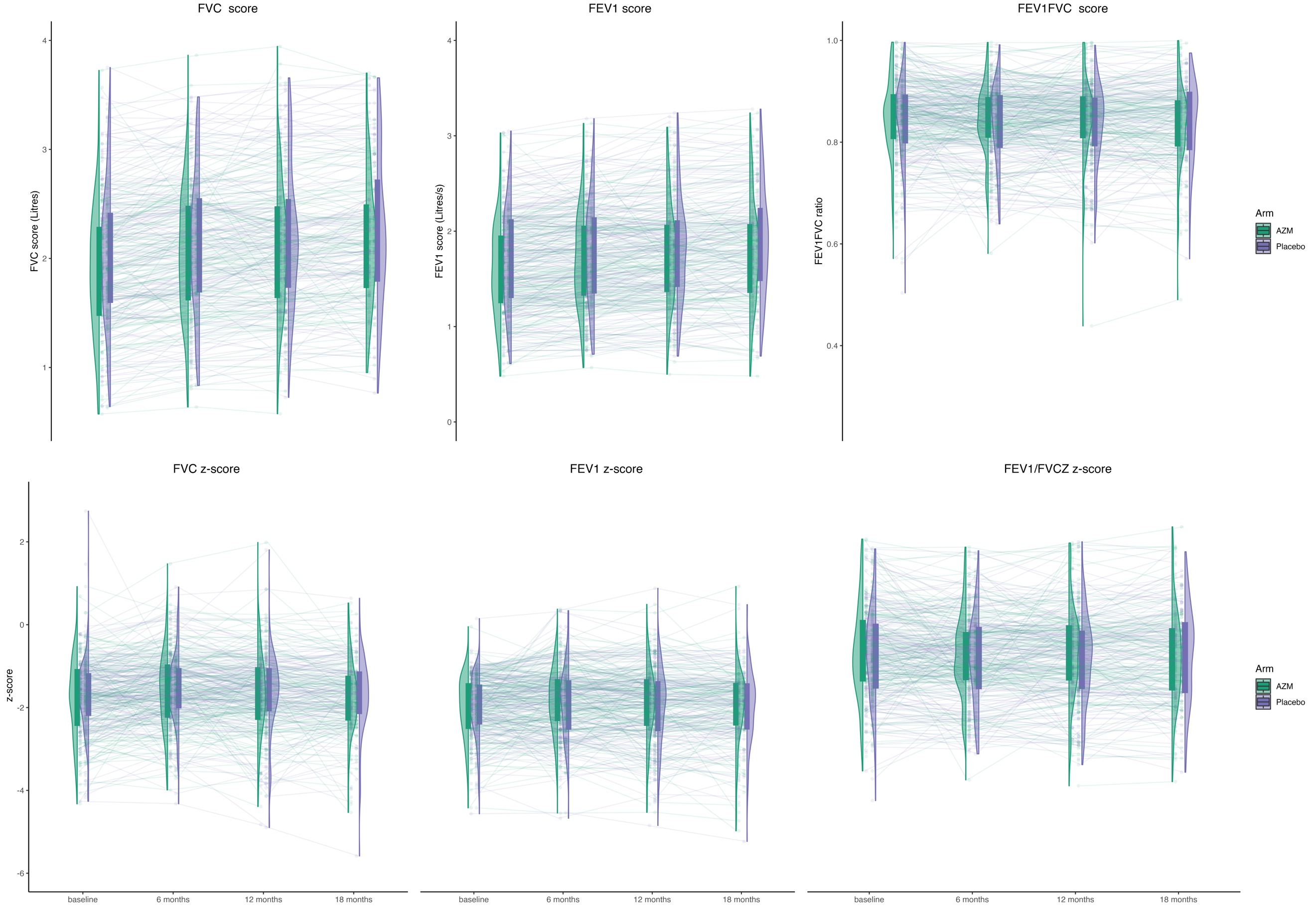

Supplement: Supplementary file 1 — Supplementary Material 1 [file 12890_2024_3155_MOESM1_ESM.docx]
