# Supplementary material for: Longitudinal lung function trajectories in response to azithromycin therapy for chronic lung disease in children with HIV infection: a secondary analysis of the BREATHE trial
Source: BMC Pulm Med. 2024 Jul 12;24:339. doi: 10.1186/s12890-024-03155-x (PMC11245797; doi:10.1186/s12890-024-03155-x)
Supplement: Supplementary file 2 — Supplementary Material 2 [file 12890_2024_3155_MOESM2_ESM.docx]

Supplementary table 1: Baseline characteristics of participants who lost to follow up and those who completed the study

| **Variables** | **Completed**  (n=248) | **Lost to follow-up**  (n=99) | **p-value** |
| --- | --- | --- | --- |
| **Demographic characteristics, n (%)** | | |  |
| Age categories at baseline  <11 years  11-15 years  ≥16 years | 33 (13.3)  112 (45.2)  103 (41.5) | 14 (14.1)  48 (48.5)  37 (37.4) | 0.787 |
| Sex  Male  Female | 133 (53.6)  115 46.4) | 44 (44.4)  55 (55.6) | 0.122 |
| Country  Malawi  Zimbabwe | 63 (25.4)  185 (74.6) | 43 (43.4)  56 (56.7) | 0.001 |
| **Clinical history** | | |  |
| Viral load (<1000 copies/ml), n (%) | 141 (56.9) | 53 (53.4) | 0.075 |
| Duration taking ART, median (IQR) | 6.8 (4.4-8.9) | 5.7 (3.7-8.5) | 0.141 |
| **Anthropometry, n (%)** | | |  |
| Height for age (z-score <-2) | 125 (50.4) | 50 (50.5) | 0.987 |
| Weight for age (z-score <-2) | 136 (54.8) | 45 (45.5) | 0.113 |
| **Spirometry, mean (SD)** | | |  |
| FEV_1_ | 1.66 (0.54) | 1.64 (0.46) | 0.670 |
| FEV_1_ z-score | -2.04 (0.76) | -1.91 (0.71) | 0.134 |
| FVC, | 1.97 (0.63) | 1.93 (0.56) | 0.518 |
| FVC z-score | -1.76 (0.93) | -1.68 (0.92) | 0.447 |
| FEV_1_/FVC | 0.85 (0.08) | 0.86 (0.08) | 0.288 |
| FEV_1_/FVC z-score | -0.77 (1.14) | -0.53 (1.11) | 0.086 |
| **Severity of lung disease, n (%)** | | |  |
| FEV_1_ z-score (<-2) | 115 (46.4) | 40 (40.4) | 0.313 |
| FVC z-score (<-2) | 81 (32.7) | 34 (34.4) | 0.764 |
| FEV_1_/FVC z-score (<-2) | 33 (13.3) | 7 (7.1) | 0.103 |
| Resistance to AZM | 24 (9.7) | 11 (11.1) | 0.689 |

*AZM: Azithromycin; IQR: Interquartile range; 4 and 2 missing values for FVC and viral load respectively*

## Supplementary table 2: Number of Adverse Events

| **Event** | **AZM arm n=173** | **Placebo arm n=174** |
| --- | --- | --- |
| **Adverse events** |  |  |
| Any adverse event | 96 | 93 |
| Any serious adverse event*^a^* | 3 | 17 |
| Any event leading to discontinuation of trial drug*^b^* | 3 | 4 |
| **Trial drug related: DAIDS grading** |  |  |
| 1 | 46 | 19 |
| 2 | 4 | 2 |
| 3 | 0 | 0 |
| 4 | 0 | 0 |
| 5 | 0 | 0 |
| **Not trial drug related: DAIDS grading** |  |  |
| 1 | 36 | 46 |
| 2 | 8 | 10 |
| 3 | 2 | 12 |
| 4 | 0 | 2 |
| 5 | 0 | 2 |

*Serious adverse event defined as life threatening, resulting in hospitalization or death*

*^a^ Placebo arm: Meningo-encephalitis n=3, Anemia n=3, Pulmonary tuberculosis n=2, Esophageal candidiasis n=2, vomiting n=1, weight loss n=4, hyperthyroidism n=1, Efavirenz induced neuropsychiatric symptoms n=1;*

*^a^ AZM arm: Herpes simplex infection n=1, Gastritis n=1, Anemia n=1*

*^b^ Placebo arm: Prolonged QTc interval n=2, pregnancy n=2;*

*^b^AZM arm: Prolonged QTc interval n=1, Pregnancy n=1, skin rash n=1*
